# Correlation Between Age and Hormone Receptor Status in Women With Breast Cancer

**DOI:** 10.7759/cureus.21652

**Published:** 2022-01-26

**Authors:** Aamera Shah, Ghulam Haider, Nargis Abro, Sorath Bhutto, Tooba I Baqai, Saba Akhtar, Kiran Abbas

**Affiliations:** 1 Department of Oncology, Jinnah Postgraduate Medical Centre, Karachi, PAK; 2 Department of Medicine, Jinnah Postgraduate Medical Centre, Karachi, PAK

**Keywords:** health, women, breast cancer, her/2neu expression, pr, er, hormonal receptor status, age

## Abstract

Introduction

Breast cancer is a leading cause of death among women. This study aimed to evaluate the association between age and hormonal receptor status (HRS) in women with breast cancer presented at a public hospital in Karachi, Pakistan.

Methods

A cross-sectional study was conducted at the Department of Medical Oncology, Jinnah Postgraduate Medical Center (JPMC), Karachi, Pakistan, from January 2021 to August 2021. All women of age more than 18 years with a confirmed diagnosis of breast cancer were included in the study using non-random consecutive sampling techniques. Women who underwent artificial menopause or hysterectomy, women who had chemotherapy-induced menopause, and pregnant women were excluded from the study. Data were collected from all patients regarding socio-demographics and tumor characteristics. Immunohistochemistry (IHC) was performed to evaluate the status of hormonal receptors.

Results

The mean age at the time of presentation of females with breast cancer was 46.57±11.45 years. Among 317 females, 180 females had positive estrogen receptor (ER) expression (56.8%), 173 had positive progesterone receptor (PR) expression (54.6%), and 121 had positive human epidermal growth factor receptor 2 (HER2/neu) expression (38.2%). The highest proportions of positive ER (36.7%), PR (38.2%), and HER/2 neu (37.2%) expression were observed in the age group 41-50 years, respectively. There was a statistically significant association between age and ER expression (p=0.017) and age and PR expression (p=0.003) while no association was found between age and HER/2 neu expression (p=0.335).

Conclusion

The present study indicated that the majority of the patients were diagnosed with breast cancer in their 40s. Most of the women in the younger age groups were estrogen receptor (ER), progesterone receptor (PR), and HER2/neu negative while the older aged women were more frequently ER, PR, and HER2/neu positive albeit, the association between age or HER2/neu was not significant.

In short, we can expect that the older aged patients may have better survival rates and patient prognosis. However, this is just a conjecture and further large-scale, multicenter, and long-term studies are required to understand the true relationship between age and patient survival rates. We hope that the current study will serve as a catalyst for future breast-cancer related studies.

## Introduction

Breast cancer is the most frequent cancer in women worldwide, affecting one out of every eight women [1]. It accounts for 16% of all female malignancies and 25% of all invasive cancers in females [2]. According to the World Health Organization (WHO) in 2020 worldwide, nearly 2.3 million females were diagnosed with breast cancer and 685,000 mortalities occurred due to it. Breast cancer occurs in every country of the world in women at any age after puberty but with increasing rates in later life [3]. In Western Europe and the United States, the mean age at the time of presentation is 63 years; however, in Asian and Arab countries [4], the mean age at the time of diagnosis is lower, such as 48.8 years in Saudi Arabia, 51.92 years in South India, and 47.5 years in Pakistan [2,5,6]. 

Breast cancer is more aggressive and advanced in younger women than in older women [7]. Evidence suggests that younger females are prone to more aggressive breast cancer than elder women. Younger age has been associated with negative hormone receptor cancers, have higher grades, and often poorly differentiated. Furthermore, age has also been shown in relation to the overexpression of human epidermal growth factor receptor 2 (HER2/neu) which indicates a worse prognosis [7,8]. 

Treatment decisions for breast cancer are commonly influenced by factors, such as age, tumor size, menopausal status, axillary nodal status, HER2/neu expression, and hormone receptor status [8]. The relationship between hormone receptor status and age in Pakistani women, particularly those from low-income families with low education, is unclear. Khan et al. revealed that local ethnic groups in Pakistan were significantly associated with hormone receptor status [9]. However, not much local literature is available on the relationship between age and hormone receptor status. Therefore, the current study was undertaken to observe the effect of age on the hormone receptor status of women with breast cancer who presented to a public hospital in Karachi, Pakistan.

## Materials and methods

A cross-sectional study was conducted at the Department of Medical Oncology, Jinnah Postgraduate Medical Centre (JPMC), Karachi, Pakistan, from January 2021 to August 2021. A non-probability convenient sampling technique was used to recruit participants in the study. The sample size was estimated using the WHO sample size calculator by taking statistics of progesterone receptor (PR) positivity among older women with breast cancer as 53.2%, absolute precision as 5.5%, and 95% confidence level [2]. The estimated sample size came out as 317.

All women of age more than 18 years with a confirmed diagnosis of breast cancer were included in the study using non-random consecutive sampling techniques. Women who underwent artificial menopause or hysterectomy, women who had chemotherapy-induced menopause, and pregnant women were excluded from the study. Ethical approval was obtained prior to the data acquisition from the institutional review board with reference # F.2-81/2021-GENL/61343/JPMC. Informed consent was obtained from all the eligible participants.

Data were collected from all patients regarding socio-demographics like age, gender, socioeconomic status, education level, employment status, residence, ethnicity, hormonal replacement, use of contraceptive pills, menopausal status, family history of any cancer, history of breast cancer, and tumor characteristics. Immunohistochemistry (IHC) was performed to evaluate the status of hormonal receptors. Patients were stratified according to the lymph node status, stage of the tumor, type of breast cancer, laterality, tumor location, and hormone receptor status (HRS) (estrogen, progesterone, HER2/neu). The cases were divided into four main groups according to the molecular subtypes of breast cancer including (i) luminal A, (ii) luminal B, (iii) triple-negative or basal-like, and (iv) HER2/neu. All breast cancer cases reflecting hormone-receptor positivity (i.e., estrogen-receptor and/or progesterone-receptor positive), HER2/neu negative, and have decreased ratio of ki-67 protein were labeled as "luminal A subtype." All hormone-receptor-positive cases with HER2/neu negativity or positivity and high levels of ki-67 were regarded as "luminal B subtype." "Triple-negative" breast cancer had both negative hormone-receptor status and HER2/neu, however, all cases where hormone-receptor status was negative but HER2/neu was positive were defined as "HER/neu subtype."

All data were analyzed using a Statistical Package for Social Sciences (SPSS) Version 26 (Chicago, IL: IBM Corp.). All categorical variables like gender, grade, and HRS status were presented as frequency and percentages while all continuous variables like age were presented as mean and standard deviation. The association of age with HRS and molecular subtype was explored using the chi-square test. A p-value of < 0.05 was set as statistical significant. 

## Results

The mean age at the time of presentation of females with breast cancer was 46.57 ± 11.45 years and the mean age at menarche was 12.10 ± 0.91 years (Table 1). Most of the females were residents of urban areas (75.7%), Urdu speakers (48.3%), illiterate (59%), and married (89.3%). About 80.8% of the females had a positive history of ever breastfeeding, 4.4% had a hormonal replacement, 3.5% used contraceptive pills, and 58% were postmenopausal. Of 317 females, 18.3% had a positive family history of any cancer and 8.2% had a positive family history of breast cancer (Table 2).

**Table 1:** Mean age at the time of presentation and menarche of females with breast cancer

Variables		Mean ± SD
Age (years)	At presentation	46.17 ± 11.12
	At menarche	12.10 ± 0.91

**Table 2:** Baseline characteristics of the study sample (n=317)

Variables		n (%)
Residence	Rural	77 (24.3%)
	Urban	240 (75.7%)
Ethnicity	Urdu	153 (48.3%)
	Sindhi	61 (19.2%)
	Punjabi	49 (15.5%)
	Pashto	25 (7.9%)
	Baloch	24 (7.6%)
	Other	5 (1.6%)
Education level	Illiterate	187 (59%)
	Primary	93 (29.3%)
	Matric	19 (6%)
	Intermediate	9 (2.8%)
	Graduate	7 (2.2%)
	Postgraduate	2 (0.6%)
Employment status	Unemployed	291 (91.8%)
	Employed	26 (8.2%)
Marital status	Married	283 (89.3%)
	Unmarried	34 (10.7%)
History of ever breastfeed	Yes	256 (80.8%)
	No	61 (19.2%)
Hormonal replacement	Yes	14 (4.4%)
	No	303 (95.6%)
Use of contraceptive pills	Yes	11 (3.5%)
	No	306 (96.5%)
Menopausal status	Premenopausal	133 (42%)
	Postmenopausal	184 (58%)
Family history of any cancer	Yes	58 (18.3%)
	No	259 (81.7%)
Family history of breast cancer	Yes	26 (8.2%)
	No	291 (91.8%)

Almost 76.3% of the females had tumor size of 2-5 cm, 67.2% had moderately differentiated tumors, and 82.6% had positive lymph node status. The majority of the patients, i.e., 243 (76.7%) had an advanced stage of the tumor (III-IV). Out of 317 females, 91.2% had to infiltrate ductal carcinoma, 54.3% had right-sided involvement, and 69.7% had tumors at the upper outer quadrant. Among 317 females, 180 females had positive ER expression (56.8%), 173 had positive PR expression (54.6%), and 121 had positive HER2/neu expression (38.2%) (Table 3). Almost half of the patients, i.e., 160 (50.5%) were both ER and PR positive. 

**Table 3:** Clinical and histopathological characteristics of patients (n=317)

Tumor clinical characteristics		N (%)
Tumor size	0-2 cm	26 (8.2%)
	2-5 cm	242 (76.3%)
	> 5cm	49 (15.5%)
Grade of tumor	Well-differentiated	14 (4.4%)
	Moderately-differentiated	213 (67.2%)
	Poorly differentiated	90 (28.4%)
Lymph node status	Positive	262 (82.6%)
	Negative	55 (17.4%)
Stage of tumor	I	7 (2.2%)
	II	63 (19.9%)
	III	180 (56.8%)
	IV‎	67 (21.1%)
Type of breast cancer	Infiltrating lobular carcinoma	25 (7.9%)
	Infiltrating ductal carcinoma	289 (91.2%)
	Medullary carcinoma	3 (0.9%)
Laterality	Left	140 (44.2%)
	Right	172 (54.3%)
	Bilateral	5 (1.6%)
Tumor location	Upper inner quadrant	34 (10.7%)
	Lower inner quadrant	44 (13.9%
	Upper outer quadrant	221 (69.7%
	Lower outer quadrant	14 (4.4%)
	Central	4 (1.3%)
Hormone receptor status	Estrogen (ER) Positive	180 (56.8%)
	Progesterone (PR) Positive	173 (54.6%)
	HER2/neu Positive	121 (38.2%)

The highest proportions of positive ER (23.4%), PR (23.4%), and HER/2neu (16%) expressions were observed in the age group 41-50 years. There was a statistically significant association between age and ER expression (p=0.017) and age and PR expression (p=0.003) while no association was found between age and HER2/neu expression (p=0.335) (Table 4).

**Table 4:** Association of age and hormonal receptor status in women with breast cancer (n=317) ER: estrogen receptor; PR: progesterone receptor

Hormonal receptor status		20-30 years	31-40 years	41-50 years	51-60 years	61-70 years	71-80 years	p-Value
ER	Positive	14 (18.7%)	53 (18.2%)	66 (23.4%)	38 (16.9%)	7 (11.1%)	3 (20%)	0.017
	Negative	11 (14.7%)	45 (15.4%)	28 (9.9%)	37 (16.4%)	14 (22.2%)	2 (13.3%)	
PR	Positive	13 (17.3%)	51 (17.5%)	66 (23.4%)	34 (15.1%)	6 (9.5%)	3 (20%)	0.003
	Negative	12 (16%)	46 (15.8%)	28 (9.9%)	41 (18.2%)	15 (23.8%)	2 (13.3%)	
HER2/neu	Positive	8 (10.7%)	33 (11.3%)	45 (16%)	26 (11.6%)	8 (12.7%)	1 (6.7%)	0.335
	Negative	17 (22.7%)	64 (21.9%)	49 (17.4%)	49 (21.8%)	13 (20.6%)	4 (26.7%)	

The high proportions of luminal A (42.9%) and luminal B (31.1%) subtype were observed in the age group 41-50 years, whereas high proportions of HER2/neu type (32.8%) and triple-negative (35.8%) breast cancer were observed in the age group 31-40 years. However, there was no significant association between age and molecular subtype of breast cancer (Table 5).

**Table 5:** Association of age and molecular subtype in women with breast cancer (n=317) HER2/neu: human epidermal growth factor receptor 2

Age groups	Luminal A	Luminal B	HER2/neu type	Triple-negative	p-Value
20-30 years	3 (4.3%)	11 (9%)	5 (8.6%)	6 (9%)	0.291
31-40 years	17 (24.3%)	37 (30.3%)	19 (32.8%)	24 (35.8%)	
41-50 years	30 (42.9%)	38 (31.1%)	13 (22.4%)	13 (19.4%)	
51-60 years	16 (22.9%)	28 (23%)	17 (29.3%)	14 (20.9%)	
61-70 years	3 (4.3%)	6 (4.9%)	4 (6.9%)	8 (11.9%)	
71-80 years	1 (1.3%)	2 (1.6%)	0	2 (3%)	

## Discussion

Literature has revealed that the young population is less likely to be affected by breast cancer [10,11]. However, the outcomes in younger age groups remain unfavorable [12]. Multiple studies have revealed that young age breast cancer tends to be more poorly differentiated than breast cancer developed in older age [13-15]. Hence, the prognosis and management of breast cancer are age-dependent [16]. Therefore, the present study aimed to assess the effect of age on hormone receptor status (HRS) in women with breast cancer. Our study showed that the majority of the tumors were of 2-5 cm, moderately differentiated having positive lymph node status. On the contrary, a survey of the US population of females having ER and PR positive breast cancer had the most common size < 2 cm and moderately differentiated [17]. Also, studies showed the mean age of 51 years in the Pakistani population, 61 years in the US population, and > 44 years in the Iranian population [18-20]. 

Our study revealed that there was a significant association between age and ER and PR status. Age between 41 and 50 years was significantly associated with ER and PR status whereas there was no significant association with HER2/neu status. The results of the present study are in contradiction with a study that determined the effect of young age on hormone receptors. The results showed that there was no significant association of age with ER and PR [21]. Another study discovered significant differences between young (27-39 years) and older (40-80 years) ages. Females aged between 27 and 39 years presented with PR-positive breast cancer [8]. The difference might be because of the smaller sample size, racial variation, and different age groups. Additionally, our study results supported the fact that tumors developing in each age group are biologically different and warrant further research in this context. We recommend finding the effect of age on HRS-specific treatment outcomes.

Another strong aspect of our study was to determine the association between age and molecular subtype. The molecular subtype of breast cancer affects the survival rate of breast cancer patients [22]. Our study found luminal A as the most common subtype which is comparable to multiple studies [23,24]. Our study found no significant association between age and subtypes. The results are in contradiction to a study that found a significant association between subtypes and increased age [25]. 

These results differ because of genetic variation, racial discrimination, staging of disease, and age groups. We recommend investigating the effect of age on different subtypes with a larger sample size and assessing age-specific effects on treatment outcomes. There are certain limitations of our study. For instance, the generalizability of our results is still limited due to the small sample size. However, it has underpinned a new aspect of breast cancer that emphasizes further research. Also, we recommend tailoring age-specific treatments depending upon different subtypes and HRS. 

## Conclusions

The present study indicated that the majority of the patients were diagnosed with breast cancer in their 40s. Most of the women in the younger age groups were estrogen receptor (ER), progesterone receptor (PR), and HER2/neu negative while the older aged women were more frequently ER, PR, and HER2/neu positive albeit the association between age and HER2/neu was not significant. 

In short, we can expect that the older aged patients may have better survival rates and patient prognosis. However, this is just a conjecture and further large-scale, multicenter, and long-term studies are required to understand the true relationship between age and patient survival rates. We hope that the current study will serve as a catalyst for future breast-cancer related studies.
